# The Role of ADF/Cofilin in Synaptic Physiology and Alzheimer’s Disease

**DOI:** 10.3389/fcell.2020.594998

**Published:** 2020-11-12

**Authors:** Youssif Ben Zablah, Neil Merovitch, Zhengping Jia

**Affiliations:** ^1^Program in Neurosciences and Mental Health, The Hospital for Sick Children, Peter Gilgan Centre for Research and Learning, Toronto, ON, Canada; ^2^Department of Physiology, Temerty Faculty of Medicine, University of Toronto, Toronto, ON, Canada

**Keywords:** ADF/cofilin, dendritic spine, LTP, LTD, AMPA glutamate receptor

## Abstract

Actin-depolymerization factor (ADF)/cofilin, a family of actin-binding proteins, are critical for the regulation of actin reorganization in response to various signals. Accumulating evidence indicates that ADF/cofilin also play important roles in neuronal structure and function, including long-term potentiation and depression. These are the most extensively studied forms of long-lasting synaptic plasticity and are widely regarded as cellular mechanisms underlying learning and memory. ADF/cofilin regulate synaptic function through their effects on dendritic spines and the trafficking of glutamate receptors, the principal mediator of excitatory synaptic transmission in vertebrates. Regulation of ADF/cofilin involves various signaling pathways converging on LIM domain kinases and slingshot phosphatases, which phosphorylate/inactivate and dephosphorylate/activate ADF/cofilin, respectively. Actin-depolymerization factor/cofilin activity is also regulated by other actin-binding proteins, activity-dependent subcellular distribution and protein translation. Abnormalities in ADF/cofilin have been associated with several neurodegenerative disorders such as Alzheimer’s disease. Therefore, investigating the roles of ADF/cofilin in the brain is not only important for understanding the fundamental processes governing neuronal structure and function, but also may provide potential therapeutic strategies to treat brain disorders.

## Introduction

Long-lasting changes in the efficacy of synaptic transmission, including long-term potentiation (LTP) and depression (LTD), are widely regarded as the key mechanisms underlying memory storage (Bliss and Collingridge, 1993; Malenka and Bear, 2004; Citri and Malenka, 2008; Neves et al., 2008; Kessels and Malinow, 2009; Kandel et al., 2014; Mateos-Aparicio and Rodríguez-Moreno, 2019). Synaptic plasticity involves changes in postsynaptic reorganization, including glutamate receptor trafficking and morphological remodeling of dendritic spines (Malinow and Malenka, 2002; Bredt and Nicoll, 2003; Collingridge et al., 2004; Lamprecht and LeDoux, 2004; Carlisle and Kennedy, 2005; Segal, 2005; Alvarez and Sabatini, 2007; Ho et al., 2011; Huganir and Nicoll, 2013; Henley and Wilkinson, 2016; Diering and Huganir, 2018), both of which are regulated by the actin cytoskeleton (Cingolani and Goda, 2008; Spence and Soderling, 2015; Nakahata and Yasuda, 2018). Evidence suggests that actin-binding proteins are involved in receptor trafficking as well as morphological changes at the synapse and consequently affect learning and memory (Lamprecht, 2011, 2016; Spence and Soderling, 2015; Borovac et al., 2018). Abnormalities in these proteins are associated with several neurological disorders (Lian and Sheen, 2015). In this review we will focus on the role of actin-depolymerization factor (ADF)/cofilin in the regulation of LTP, LTD, dendritic spines and their dysfunction in Alzheimer’s disease (AD).

## Actin-Depolymerizing Proteins

Cofilin is a member of the actin-depolymerizing protein family that is important for the regulation of actin cytoskeleton dynamics (Ridley, 2011; Kanellos and Frame, 2016). This family includes cofilin-1 (n-cofilin, non-muscle), cofilin-2 (muscle cofilin) and actin-depolymerization factor (ADF, destrin) and is well conserved among eukaryotes (Maciver and Hussey, 2002). These proteins have a molecular mass of 15–19 kDa and share multiple structural similarities. Each consists of an actin depolymerizing factor homology (ADF-H) domain, which allows for binding to actin subunits, a central alpha helix, a N-terminus extension and a C-terminus helix (Lappalainen et al., 1998; Shishkin et al., 2016). Despite their similarities at the molecular level, these isoforms differ in their degree of affinity for actin (Bamburg, 1999; Vartiainen et al., 2002; Yeoh et al., 2002). Actin-depolymerization factor and cofilin-1 can bind to actin filaments with similar degrees of affinity, whereas cofilin-2 is less efficient at depolymerization (Vartiainen et al., 2002). While ADF is better at sequestering monomeric actin, cofilin-1 is more efficient at nucleation and severing actin filaments (Chin et al., 2016). These biochemical differences reflect variations in the cellular expression between isoforms, where ADF and cofilin-1 are mainly expressed in tissues with higher actin turnover. Specifically, while cofilin-1 is expressed in all cell types, ADF is mainly expressed in neuronal, epithelial, and endothelial cells (Kanellos and Frame, 2016). Cofilin-2 is also expressed in selected tissues including muscles and brain (Thirion et al., 2001; Agrawal et al., 2012; Gurniak et al., 2014). This review will discuss ADF and cofilin-1, which are expressed in neuronal cells. Many studies addressing the roles of ADF/cofilin do not specify which isoform as many of their functions overlap. Also, most antibodies do not differentiate between these isoforms and rescue experiments often use cofilin from lower level eukaryotes that express only one isoform (Moon et al., 1993; Kanellos and Frame, 2016). For these reasons and the sake of simplicity, this group of actin depolymerizing factors will be referred to collectively as ADF/cofilin, except in studies where specific isoforms have been addressed.

## General Cellular Function of ADF/Cofilin

### Regulation of Actin Dynamics

**FIGURE 1:**
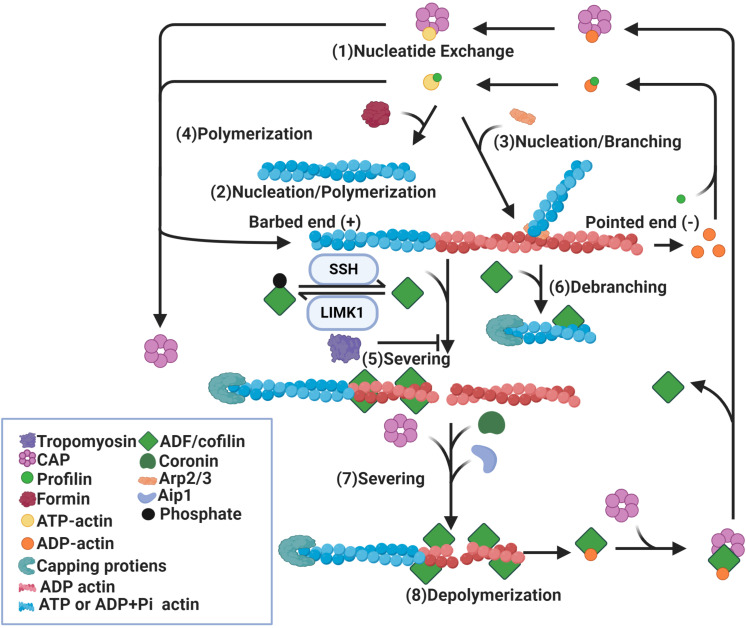
Regulation of actin dynamics by ADF/cofilin. Binding of profilin on ADP-actin monomers induces nucleotide exchange (1). Formin (2) and Arp2/3 (3) induce nucleation of actin monomers and formation of actin filaments. While formin induces parallel actin filament (2), Arp2/3 promotes branching of the original filament (3). In addition, actin filaments can be polymerized by the addition of ATP-actin monomers at the barbed ends (4). Binding of dephosphorylated/active ADF/cofilin to ADP-actin subunits of actin filaments causes severing of these filaments (5) and depolymerization at pointed ends (8). ADF/cofilin activity is mediated by phosphorylation and dephosphorylation by LIMK1 and SSH respectively. ADF/cofilin also debranch Arp2/3 nucleated actin filaments (6). The severing activity of ADF/cofilin is enhanced by Aip1, coronin and CAP (7) and diminished by tropomyosin (5). Binding of capping proteins at barbed ends blocks the growth of newly formed actin segments. CAP also dissociates ADF/cofilin from ADP-actin monomers and promotes nucleotide exchange on these monomers (1).

The most characterized role of ADF/cofilin is the regulation of actin reorganization and their capacity to increase actin filament turnover (Pollard and Borisy, 2003; Brieher, 2013). Treadmilling is the most accepted model for actin turnover (Figure 1; Wegner, 1982; Blanchoin et al., 2014). In this model, steady state actin filaments preferentially grow at one end, known as the barbed end, by association of ATP-bound actin monomers, whereas actin monomers dissociate at the other end, known as the pointed end (Pollard and Borisy, 2003; Lee and Dominguez, 2010). Following the addition of the ATP-bound actin subunit, ATP undergoes hydrolysis into ADP and Pi, after which Pi is released, leaving ADP-bound subunits at the pointed end. ADP-bound subunits are more prone to dissociate and return to the actin monomers pool. Dissociated ADP-bound subunits then exchange ADP into ATP before entering the cycle again (Carlier and Pantaloni, 1986; Blanchoin and Pollard, 2002; Pollard and Borisy, 2003). The dynamic turnover of actin filaments can be enhanced by an increase in the number of filament ends due to severing of existing filaments (Ichetovkin et al., 2000; Pavlov et al., 2007). It can also be enhanced by an increase in the rate of association (polymerization or nucleation) at the barbed ends and dissociation (depolymerization) at pointed ends (Carlier et al., 1997*;* Kiuchi et al., 2007).

Multiple studies show a significant role for ADF/cofilin in actin filament assembly and disassembly (Lappalainen and Drubin, 1997; Rosenblatt et al., 1997; Loisel et al., 1999; Pollard and Borisy, 2003; Bernstein and Bamburg, 2010; Kanellos and Frame, 2016). Two models have been proposed to explain the disassembly function of ADF/cofilin. Actin-depolymerization factor/cofilin can increase the rate of depolymerization at pointed ends or sever existing actin filaments into smaller fragments (Blanchoin and Pollard, 1999; Pavlov et al., 2007). The best evidence for increased actin subunit dissociation comes from the “bulk sample of actin” experiment that measured the exchange of fluorescent or radiolabeled ADP-bound actin subunits incubated with ADF/cofilin for ATP in the medium (Carlier et al., 1997). Exchange of ADP into ATP occurs only on free actin monomers not on actin subunits in filaments, therefore nucleotide exchange can only happen after the dissociation of ADP-bound subunits from filaments. The observed increase in the rate of nucleotide exchange in this experiment can be interpreted to arise from the dissociation of actin subunits in the presence of ADF/cofilin (Carlier et al., 1997). Though this study suggests that depolymerization at the pointed end increases in the presence of ADF/cofilin, it does not provide direct evidence to support this conclusion. As neither the number of ends nor filament lengths were known, it was not possible to measure subunit dissociation from individual filament ends (Carlier et al., 1997). The filament disassembly severing model is supported by real-time microscopy assays which analyzed single actin filaments. Binding of ADF/cofilin to actin filaments was found to induce a conformational twist in these filaments resulting in fragmentation or severing of filaments (Andrianantoandro and Pollard, 2006). After binding of ADF/cofilin, an increase in actin depolymerization at pointed ends was observed in the presence of vitamin D binding proteins, which sequester free actin monomers. However, the detected rate of depolymerization was too slow to account for the observed rates of nucleotide exchange in bulk assays (Andrianantoandro and Pollard, 2006). Therefore, it could be concluded that severing of actin filaments by ADF/cofilin can produce many filament ends which may account for the nucleotide exchange rate (Ichetovkin et al., 2000; Pavlov et al., 2007). Recently, a study using single-filament approach based on microfluidics suggests that ADF/cofilin-induced actin disassembly is mediated by both severing and depolymerization activity (Wioland et al., 2017). Consistent with Andrianantoandro and Pollard (2006), pointed end depolymerization was enhanced by ADF/cofilin, though not to the extent predicted by bulk assays (Andrianantoandro and Pollard, 2006; Wioland et al., 2017). Actin-depolymerization factor/cofilin favor barbed-end depolymerization through either directly targeting the barbed ends of bare filaments, which is avoided when ATP-actin is present, or preventing the barbed ends of ADF/cofilin-saturated filaments from elongating and promoting barbed-end depolymerization, contrary to the general consensus (Wioland et al., 2017). As there is debate on how ADF/cofilin promote filament disassembly, they also promote assembly in multiple ways. Actin-depolymerization factor/cofilin could increase the rate of polymerization as detected in bulk assays (Carlier et al., 1997). However, single filament studies show that ADF/cofilin slow barbed-end polymerization (Andrianantoandro and Pollard, 2006). Another mechanism for cofilin-induced filament assembly is through severing, which may create more filament ends (Andrianantoandro and Pollard, 2006). Moreover, cofilin could stimulate nucleation by stabilizing long-pitch actin dimers, the first intermediate in spontaneous assembly and nucleation may be the main contribution of ADF to the increased rate of actin filament assembly (Andrianantoandro and Pollard, 2006). In summary, ADF/cofilin mediate both filament assembly and disassembly through multiple mechanisms, including depolymerization, severing, polymerization and nucleation (Figure 1).

The effect of ADF/cofilin on actin filaments depends on the relative concentration of ADF/cofilin to actin and interactions with other proteins (Ono, 2003; Winder and Ayscough, 2005; Pavlov et al., 2007). At a lower ADF/cofilin concentration, severing of actin filaments by ADF/cofilin is highest (Andrianantoandro and Pollard, 2006). When few ADF/cofilin molecules are bound to actin filaments, the number of strained interfaces between twisted and non-twisted region is the highest, resulting in frequent breakage (Bobkov et al., 2006). At a higher ADF/cofilin concentration, when actin filaments are largely covered with ADF/cofilin, severing is no longer observed, though there is still dissociation from the pointed ends (Pavlov et al., 2007). When ADF/cofilin levels are higher, they can nucleate filaments (Yeoh et al., 2002; Andrianantoandro and Pollard, 2006; Kudryashov et al., 2006). However, abnormally high levels of active ADF/cofilin can drive the formation of ADF/cofilin-actin rods that sequester a large fraction of the total ADF/cofilin, rendering ADF/cofilin incapable of promoting actin disassembly (Minamide et al., 2000). Other actin-binding proteins may alter ADF/cofilin’s ability to act on the actin cytoskeleton (Winder and Ayscough, 2005). These proteins include actin-interacting protein 1 (AIP1), tropomyosins (TPM), cortactin, actin-related proteins-2/3 (Arp2/3) and coronins (Ichetovkin et al., 2002; Brieher et al., 2006; Kueh et al., 2008; Ostrowska-Podhorodecka et al., 2020).

### Interaction With and Regulation of Other Actin-Binding Proteins

The reported rates of ADF/cofilin-mediated actin filament disassembly *in vitro* are lower than those observed in *in vivo* experiments, which could be due to a difference in cytosolic versus *in vitro* conditions (Lappalainen and Drubin, 1997). These results also suggest that additional cellular factors may be involved in regulating the activity of ADF/cofilin under physiological conditions. Many studies have demonstrated that other actin-binding proteins can potently modulate ADF/cofilin’s ability to act on the actin cytoskeleton (Winder and Ayscough, 2005). These actin-binding proteins include: (1) proteins structurally or functionally similar to ADF/Cofilin (e.g., AIP1), cyclase associated protein (CAP), and coronin; (2) proteins involved in F-actin filament assembly (e.g., Arp 2/3, profilin, and cortactin); (3) proteins generally antagonistic toward ADF/Cofilin activity (e.g., TPM) (Winder and Ayscough, 2005). Actin-interacting protein 1, coronin, and CAP are functionally similar to ADF/cofilin as they each promote F-actin disassembly. Both AIP1 and coronin facilitate the cofilin-mediated disassembly of Listeria comet tail and purified actin filaments even with a physiological concentration of actin monomers, a condition promoting actin assembly (Brieher et al., 2006; Kueh et al., 2008). This conclusion is supported further using internal reflection fluorescence microscopy to directly visualize the integrated actions of coronin and AIP in enhancing cofilin-mediated actin filaments disassembly (Jansen et al., 2015). Although AIP1 itself moderately enhances cofilin-mediated actin severing, the presence of coronin alone appears to inhibit severing by cofilin (Jansen et al., 2015). The inhibitory effect of coronin was also previously shown in bulk assay studies (Cai et al., 2007; Gandhi et al., 2009). This disparity is likely to arise due to differences in the nucleotides state of actin, as studies have shown that coronin may interfere with the binding of cofilin to ATP-actin, but not ADP-actin (Ge et al., 2014). In addition to the synergistic effect of coronin and AIP1 on cofilin-mediated actin severing, AIP1 has been shown to be able to bind to the newly generated barbed ends and block growth of the newly formed actin segments, enabling actin filament disassembly under cellular conditions which generally enables filament assembly (Jansen et al., 2015). Actin-interacting protein 1 is known to be regulated by STK16, a constitutive kinase. RNAi knockdown of STK16 in cultured cells resulted in significantly decreased F-actin levels and increased actin polymerization, demonstrating a potential link between AIP1 activity and actin dynamics (Liu et al., 2017). Interestingly, the mixture of cofilin, coronin and AIP1 failed to disassemble actin filaments with a physiological concentration of actin filaments until the addition of CAP (Normoyle and Brieher, 2012). As such, CAP was identified as a factor that promotes disassembly of cofilin-actin filaments. Cyclase associated protein was shown to associate with both actin monomers and filaments and is expressed in the hippocampus, striatum and cortex (Freeman et al., 1995; Bertling et al., 2004; Normoyle and Brieher, 2012). CAP1 knockdown in cultured cells results in abnormal cytoplasmic aggregates of cofilin and diminished actin depolymerization, suggesting a role of CAP in regulating the localization and function of cofilin-1 in mammalian cells (Bertling et al., 2004). Interestingly, CAP forms a hexameric structure that binds to actin filaments though its N-terminal segment and enhances cofilin-mediated actin severing (Jansen et al., 2014). The severing efficiency of CAP is directly proportional to the stoichiometry of their oligomerization, that is to say CAP tetramers and trimers show increased CAP-cofilin interaction compared to CAP monomers (Purde et al., 2019). Despite these studies suggesting a role of CAP in cofilin-mediated filament severing, recent studies report the inability of CAP to increase cofilin-mediated actin severing using single-filament microfluidics approach (Shekhar et al., 2019). In the same line, CAP accelerates actin depolymerization at the pointed end suggesting that CAP enhance cofilin-mediated disassembly through depolymerization not severing (Kotila et al., 2019; Shekhar et al., 2019). Cyclase associated protein can also bind to actin monomers through its C-terminal domain and catalyze nucleotide exchange on cofilin-bound ADP actin monomers (Jansen et al., 2014; Kotila et al., 2018). Proteins involved in F-actin assembly/nucleation shown to interact with ADF/cofilin include Arp2/3, profilin and formin (Weaver et al., 2001; Sagot et al., 2002; Bleicher et al., 2020). Early *in vitro* studies suggest a synergistic relationship between the Arp2/3 complex and cofilin in regulating filament assembly. The total number of newly polymerized filaments is increased in the presence of both the Arp2/3 complex and cofilin (Ichetovkin et al., 2002). In addition, the frequency of Arp2/3-nucleated branching, in newly formed actin filaments from cofilin-mediated severing, is higher than old pre-existing filaments. Other studies suggest cofilin promotes debranching and has an antagonistic relationship with Arp2/3 as cofilin promotes dissociation of actin filament branches induced by Arp 2/3 (Blanchoin et al., 2000). Moreover, binding of cofilin promotes structural changes in actin filaments, which decreases the affinity of Arp2/3 complex for actin resulting in dissociation of Arp2/3 complex from actin filaments and promoting dissociation of actin filament branches induced by Arp 2/3 complex (Chan et al., 2009). Therefore, the debranching activity of cofilin occurs via cofilin’s effect on actin filaments and not the Arp2/3 complex itself. However, some studies suggest that actin depolymerizing factor homology protein, known as glia maturation factor, functions more specifically as a debranching factor through direct interaction with the Arp2/3 complex (Ydenberg et al., 2013; Poukkula et al., 2014). Much like the process of disassembly, actin filament nucleation is synergistic and reliant on the concentration of both actin monomers and proteins required for polymerization. There is a certain degree of cooperativity between actin filament assembly and disassembly, as it has been shown that formin activity can preclude cofilin-mediated severing, although cofilin activity is required to produce the actin monomers needed to maintain network stability (Bleicher et al., 2020). Tropomyosin is a well characterized regulator of actin filament dynamics known to dampen ADF/cofilin activity (Gunning et al., 2015). Specifically, Tpm competes with ADF/cofilin-mediated actin disassembly by spatially restricting binding sites at the pointed ends of filaments (Kuhn and Bamburg, 2008; Gateva et al., 2017; Jansen and Goode, 2019). Notably, different Tpm isoforms have varying effects on ADF/cofilin-mediated actin dynamics; particularly, fast off-rate Tpm isoforms permit a relative increase in ADF/cofilin binding, allowing for greater F-actin turnover (Ostrowska-Podhorodecka et al., 2020). In summary, ADF/cofilin activity is intricately regulated by its interactions with diverse actin-binding proteins. Some of these proteins have been shown to play an important role in spine and synaptic plasticity and will be further discussed in later sections.

### Activation of Phospholipase D1

The phosphorylated form of ADF/cofilin is considered the inactive form and is not involved in actin cytoskeleton regulation (Bernstein and Bamburg, 2010; Ridley, 2011; Kanellos and Frame, 2016). Few studies have shown a role of phosphorylated ADF/cofilin in muscarinic receptor−mediated stimulation of phospholipase D1 (PLD1), which is independent of actin regulation (Schmidt et al., 1999; Han et al., 2007). Phosphorylated ADF/cofilin can bind and activate PLD1, leading to the hydrolysis of phosphatidylcholine to phosphatidic acid by PLD1 in the cell membrane, and is considered to be involved in a large variety of early and late cellular responses. These responses include calcium mobilization, secretion, superoxide production, endocytosis, exocytosis, vesicle trafficking, glucose transport, mitogenesis and apoptosis (Exton, 2002). In HEK-293 and neuroblastoma cells, factors known to increase ADF/cofilin phosphorylation, such as LIM domain containing kinase (LIMK) 1 and inactive slingshot phosphatase (SSH) enhance the activity of PLD1, whereas expression of wild-type SSH, which abolishes ADF/cofilin phosphorylation, and constitutively active unphosphorylatable (S3A) cofilin compromise PLD stimulation (Han et al., 2007). Phospholipase D1 activity has been linked to neurite outgrowth and LTD, suggesting its involvement in synaptic plasticity but further characterization is required (Cai et al., 2006; Santa-Marinha et al., 2020). Thus, even in its phosphorylated, presumed inactive form, ADF/cofilin is likely to fulfil important biological roles.

## Regulation of ADF/Cofilin Activity

### ADF/Cofilin Phosphorylation/Dephosphorylation

Actin-depolymerization factor/cofilin phosphorylation/dephosphorylation at serine 3 (Ser 3) serves as a key convergence point for many signaling pathways to regulate ADF/cofilin activity in response to various intrinsic and external signals (Bamburg, 1999; Bamburg and Bernstein, 2008). Actin-depolymerization factor/cofilin phosphorylation at Ser 3 inhibits actin binding, whereas dephosphorylation activates actin binding (Bernstein and Bamburg, 2010; Kanellos and Frame, 2016). Actin-depolymerization factor/cofilin Ser 3 phosphorylation is mediated by LIMK and testicular protein kinase (TESK), which are serine/threonine kinases (Arber et al., 1998; Yang et al., 1998; Toshima et al., 2001). LIMKs are extensively studied and contain two family members; LIMK1 is predominantly expressed in the nervous system and LIMK2 is widespread throughout the body (Scott and Olson, 2007; Cuberos et al., 2015). LIMK1/2 have high specificity for Ser3 of ADF/cofilin, due to the interaction between the LIMK catalytic domain and the actin binding helix of ADF/cofilin. Targeted mutations at the phosphorylation site inhibit functional inactivation of cofilin-1 by LIMK1 in yeast and mammalian cells (Hamill et al., 2016). LIMK1/2 can be phosphorylated and activated by the Rho-associated protein kinases (ROCKs) and p21-activated kinases (PAKs; Scott and Olson, 2007; Arber et al., 1998; Yang et al., 1998; Cuberos et al., 2015). Phosphorylation of LIMK1 by PAK1 and LIMK2 by PAK4 occurs at Thr 508 and 505, respectively (Maekawa et al., 1999; Ohashi et al., 2000; Sumi et al., 2001). Both PAKs and ROCKs are protein kinases associated with and activated by the Rho family of small GTPases, the central mediators of actin reorganization in response to diverse signaling processes (Govek et al., 2005). The importance of LIMK1 for ADF/cofilin phosphorylation and actin regulation is shown by reduced ADF/cofilin phosphorylation and altered F-actin in LIMK1 knockout (KO) mice (Meng et al., 2002). Cofilin dephosphorylation at Ser 3 is mediated by two protein phosphatases; chronophin, which is highly specific for cofilin, and SSH, which can also dephosphorylate and inactivate LIMK1 (Niwa et al., 2002; Bernstein and Bamburg, 2010). SSH can be phosphorylated and inactivated by PAK4 and protein kinase D1 (Soosairajah et al., 2005; Eiseler et al., 2009). Both chronophin and SSH regulate ADF/cofilin in a spatially precise manner, proximal to the membrane, this can allow for the formation of membrane protrusions (Nagata-Ohashi et al., 2004; Gohla et al., 2005; Nishita et al., 2005). For example, during lamellipodium formation, dephosphorylation of SSH induces its release from scaffolding protein 14-3-3 in the cytoplasm and its translocation on growing actin filaments to induce dephosphorylation/activation of cofilin within lamellipodium (Nagata-Ohashi et al., 2004).

### Other Regulatory Mechanisms

In addition to Ser 3, phosphorylation at tyrosine (Tyr 68) has been shown to be important for cofilin-1 regulation (Yoo et al., 2010). This regulation is unique to cofilin-1, since ADF does not have Tyr 68 (Yoo et al., 2010). In HEK cells, phosphorylation at Tyr 68 does not directly affect the actin-depolymerizing activity, however it increases ubiquitination and proteasome degradation of cofilin-1 sufficiently to reduce cofilin-1 levels and cellular distribution (Yoo et al., 2010). Oxidation has also been introduced as a mechanism for ADF/cofilin regulation (Bernstein and Bamburg, 2010; Kanellos and Frame, 2016). Under oxidative stress conditions in T cells, ADF/cofilin can undergo oxidative modification. Oxidation of the thiol groups of cysteine residues in ADF/cofilin molecules leads to the formation of both intra and intermolecular disulfide bonds which causes oxidized ADF/cofilin to interact weakly with LIMKs and this results in an increase in unphosphorylated/active ADF/cofilin (Klemke et al., 2008). Another mode of ADF/cofilin regulation is through binding to phosphatidylinositol 4,5-bisphosphate (PIP2; Bernstein and Bamburg, 2010; Kanellos and Frame, 2016). *In vitro* studies show that PIP2 directly binds to ADF/cofilin and inhibits their actin-depolymerizing activities (Yonezawa et al., 1990). This was confirmed using biochemical and spectroscopic studies showing that ADF/cofilin cluster PIP2 molecules at the membrane through their interaction with multiple PIP2 headgroups and that a small decrease in PIP2 density efficiently activated ADF/cofilin in carcinoma cells (Zhao et al., 2010). pH *in vitro* and *in vivo* is also shown to modulate mammalian ADF/cofilin activity (Bernstein et al., 2000; Pavlov et al., 2006). The *in vivo* mechanism is highlighted by the ability of cofilin to act as a cellular pH sensor, with increased activity at higher pH and that this ability involves the inhibition of cofilin activity by binding PIP2, as discussed earlier (Frantz et al., 2008). Studies in neurons have shown other regulatory mechanisms in addition to those introduced above and these include mRNA availability and translation (Feuge et al., 2019), and temporal and spatial regulation of subcellular distribution (e.g., Zhou et al., 2011; Pontrello et al., 2012; Bosch et al., 2014). These mechanisms are particularly important for the regulation of ADF/cofilin activity during spine and synaptic plasticity, which will be discussed further in later sections.

## Role of ADF/cofilin in Synaptic Function and Memory in the Brain

### Bidirectional Regulation of Spine Morphology

One of the most important features of neuronal synapses is their ability to change the strength of synaptic transmission in response to external stimuli, which is referred to as synaptic plasticity. In the mammalian central nervous system, most excitatory synapses are located on small dendritic protrusions called dendritic spines (Carlisle and Kennedy, 2005; Alvarez and Sabatini, 2007; Tønnesen and Nägerl, 2016; Gipson and Olive, 2017). Synaptic plasticity, including LTP and LTD, is closely associated with changes in the number and morphology of dendritic spines and these changes are typically referred to as structural plasticity (Lamprecht and LeDoux, 2004; Carlisle and Kennedy, 2005; Alvarez and Sabatini, 2007; Bernardinelli et al., 2014; Borovac et al., 2018; Lai and Ip, 2013; Sheppard et al., 2019). For example, using glutamate uncaging, an enlargement of dendritic spines during the induction of LTP at single spines of hippocampal CA1 pyramidal neurons is observed (Matsuzaki et al., 2004). On the other hand, the induction of LTD using low frequency stimulation is accompanied by shrinkage of dendric spines in acute hippocampal slices from neonatal rats (Zhou et al., 2004). As actin is the main cytoskeletal component of the dendritic spine, it is not surprising that actin dynamics play a key role in the regulation of spine morphology (Matus et al., 1982; Hotulainen and Hoogenraad, 2010; Miermans et al., 2017; Basu and Lamprecht, 2018). Using two-photon imaging, a dynamic pool of actin filaments is seen at the tips of spines from CA1 pyramidal neuron in rat hippocampal slices (Honkura et al., 2008). These actin filaments can be quickly treadmilled to generate an expansive force to mediate changes in spines (Honkura et al., 2008). Two-photon Forster resonance energy transfer (FRET) imaging shows that activity-dependent actin polymerization and depolymerization in dendritic spines during LTP and LTD (Okamoto et al., 2004). During synaptic plasticity, the actin cytoskeleton is highly regulated and goes through phases of polymerization and depolymerization (Bosch et al., 2014; Kim et al., 2015; Borovac et al., 2018). For example, the reorganization of actin during LTP appears to have two distinct but overlapping phases (Bosch et al., 2014). Within the first 5 min after LTP induction, there is remodeling of the actin cytoskeleton through rapid periods of actin filaments disassembly followed by periods of actin filament assembly, which result in enlargement of dendritic spines and LTP. After 5 min of LTP induction, there is a net increase in actin and newly polymerized actin filaments in the spine which result in long-term stabilization and consolidation of early synaptic changes (Bosch et al., 2014).

One of the earliest indications that ADF/cofilin is important for spine and synaptic regulation comes from studies on LIMK1/2 KO mice, which show altered spine morphology and impaired synaptic function and that these alterations are associated with a dramatic reduction in cofilin phosphorylation (Meng et al., 2002, 2004). In addition, bidirectional changes in ADF/cofilin phosphorylation and dephosphorylation can be induced rapidly by activation of glutamate receptors and signaling molecules at the synapse (Meng et al., 2002). A subsequent study using immunoelectron microscopy shows that cofilin-1 is concentrated in the shell of spines rich in dynamic actin and within postsynaptic density in the stratum radiatum of the rat hippocampus (Racz and Weinberg, 2006). More direct evidence to support cofilin function at the synapse comes from molecular and genetic manipulations of ADF/cofilin and their upstream regulators. Overexpression of constitutively active unphosphorylatable cofilin (S3A) in neurons leads to reduced spine size and immature morphology (Shi et al., 2009). The expression of constitutively inactive phosphomimetic cofilin (S3D) restores mature spine morphology (Shi et al., 2009). Longer dendritic protrusions and slower actin turnover are also observed when cofilin-1 expression is reduced using siRNA in primary hippocampal neurons (Hotulainen et al., 2009). In cofilin-1 conditional KO mice where cofilin-1 is selectively deleted in the excitatory neurons of the postnatal forebrain, increased synapse density and enlargement of dendritic spines are found in the hippocampus and these structural changes are associated with impaired late phase LTP and LTD (Rust et al., 2010). Although ADF KO mice show no deficits in spine properties or synaptic function (Görlich et al., 2011), ADF and cofilin-1 double KO mice exhibit greater changes in spine enlargement than cofilin-1 conditional KO mice, suggesting that ADF also plays a role in spine regulation (Wolf et al., 2015). Other evidence supporting the role of ADF/cofilin in basal spine properties comes from manipulations of their upstream regulators in addition to LIMK1/2 (Meng et al., 2002, 2004; Todorovski et al., 2015). These include PAK1/3 (Meng et al., 2005; Asrar et al., 2009; Huang et al., 2011), ROCK2 (Zhou et al., 2009), chronophin (Kim et al., 2016) and Rho GTPases (Nakayama et al., 2000; Li et al., 2002; Martino et al., 2013), all of which affect either spine morphology or density. For example, both PAK1/3 double and ROCK2 KO mice have reduced spine density and immature morphology and these spine changes are associated with a significant reduction in cofilin phosphorylation (Zhou et al., 2009; Huang et al., 2011). Overexpression of chronophin in mice results in the shrinkage of dendritic spines and knocking out chronophin causes dendritic spine enlargement in hippocampal neurons (Kim et al., 2016).

Actin-depolymerization factor/cofilin are not only important for basal spine morphology and density but also required for spine changes during synaptic plasticity for which the temporal and spatial regulation of ADF/cofilin appears to be particularly important (Bernstein and Bamburg, 2010; Lai and Ip, 2013; Noguchi et al., 2016; Borovac et al., 2018). In general, it has been shown that ADF/cofilin inactivation is associated with and required for actin assembly and spine enlargement and stabilization during LTP, whereas ADF/cofilin activation can drive actin filament disassembly and spine shrinkage during LTD. However, single spine imaging studies have revealed that changes in ADF/cofilin during LTP are much more complex and dynamic, exhibiting multiple phases of regulation (Bosch et al., 2014). During the initial phase (<5 min) of dendritic spine enlargement induced by glutamate uncaging at single spines, the amount of ADF/cofilin in the spine increases and this is accompanied by an increase in the amount of actin and spine enlargement (Bosch et al., 2014). This is consistent with the observation that cofilin undergoes translocation in its unphosphorylated/active form following glutamate uncaging (Noguchi et al., 2016). During a later phase (>5 min), there is sustained accumulation of phosphorylated/inactive ADF/cofilin at the base of the spine head where cofilin forms a stable complex with actin filaments (Bosch et al., 2014). These results suggest that ADF/cofilin phosphorylation is needed to retain its accumulation within spines. It is shown that wild type and constitutively inactive cofilin (S3E) accumulate in the stimulated spines for 30?min after spine enlargement following glutamate uncaging, whereas constitutively active cofilin (S3A) more rapidly diffuses away from the enlarged spine (Noguchi et al., 2016). These single spine imaging results are consistent with earlier studies using protocols to induce LTP at the global level. PAK and ADF/cofilin phosphorylation in rat hippocampal slices are found to be increased 2–7 min after theta-burst stimulation, a time window consistent with the transition from the initial to later phase (Chen et al., 2007). In cultured neurons, the levels of phosphorylated cofilin declines 5 min after the onset of chemically induced LTP, but significantly increases by 30 min (Gu et al., 2010). These studies suggest that dynamic regulation of ADF/cofilin phosphorylation and spine accumulation contributes to different phases of spine plasticity during LTP. During spine shrinkage and LTD, although the precise time course of ADF/cofilin involvement has not be investigated at single spines, ADF/cofilin dephosphorylation and spine accumulation are consistently found to be associated with and required for LTD in both cultured neurons and brain slices (Zhou et al., 2011; Pontrello et al., 2012). The mechanisms by which ADF/cofilin is regulated during LTP and LTD will be discussed further in later sections.

How changes in ADF/cofilin activity, either through spine accumulation or phosphorylation, regulate spine morphology remain unclear. However, a recent study using a fluorescent reporter to monitor membrane-proximal actin filaments (MPA) may provide new insight (Bisaria et al., 2020). In this study, it is shown that amount of MPA is lower at the front compared to the back during membrane protrusion and cell migration, and that increased cofilin activity is required for this MPA gradient and the initiation of new membrane protrusions. These results are consistent with earlier studies showing SSH can regulate ADF/cofilin in a spatially precise manner proximal to the membrane (Nagata-Ohashi et al., 2004; Soosairajah et al., 2005) and suggest that cofilin activity driven by SSH at the front is essential. The major isoform of slingshot is SSH-1L, which is only active in its cofilin dephosphorylation activity when it is bound to F-actin (Nagata-Ohashi et al., 2004; Soosairajah et al., 2005). In contrast to the accumulation of SSH1L, LIMK1 diffusely distributes in the cytoplasm (Nagata-Ohashi et al., 2004; Nishita et al., 2005). These findings suggest that spatially distinct localization of LIMK1 and SSH1L during protrusions formations may also play a role in spine formation and morphological changes.

### Bidirectional Regulation of Glutamate Receptors Trafficking

While the induction of LTP and LTD at many synapses requires the activation of *N*-Methyl-D-aspartic acid (NMDA) glutamate receptors, their expression involves modification of α-amino-3-hydroxy-5-methyl-4-isoxazolepropionic acid (AMPA) glutamate receptors, the principal mediator of fast excitatory synaptic transmission (Bliss and Collingridge, 1993; Malinow and Malenka, 2002; Bredt and Nicoll, 2003; Collingridge et al., 2004, 2010; Segal, 2005; Derkach et al., 2007; Rebola et al., 2010; Ho et al., 2011; Huganir and Nicoll, 2013; Henley and Wilkinson, 2016; Diering and Huganir, 2018). These modifications include channel properties and receptor abundance at the synapses (Lau and Zukin, 2007; Huganir and Nicoll, 2013; Henley and Wilkinson, 2016; Diering and Huganir, 2018). In particular, receptor trafficking at the synapse attracts the most attention due to its potent effects on synaptic strength (Collingridge et al., 2004; Henley and Wilkinson, 2016; Diering and Huganir, 2018; Park, 2018). Several studies have shown that ADF/cofilin play an important role in the regulating trafficking and accumulation of AMPA receptors within synapses during LTP and this process appears to be distinct from its role in spine morphological plasticity (Gu et al., 2010; Rust et al., 2010). Elevated ADF/cofilin activity markedly enhances addition of AMPARs to the surface after chemical induction of LTP in cultured neurons, whereas the inhibition of ADF/cofilin activity suppresses the addition of AMPA receptors (Gu et al., 2010). The role of ADF/cofilin in AMPA receptor trafficking has also been demonstrated in animal models. Lateral diffusion of the AMPA receptors subunit GluA2 was shown to be compromised in the extrasynaptic compartment of hippocampal neurons from cofilin-1 mutant mice (Rust et al., 2010). The exchange of AMPA receptors between synaptic and extrasynaptic domains by lateral diffusion is thought to represent a key mechanism to control the level of synaptic AMPA receptors during synaptic plasticity (Choquet and Hosy, 2020; Groc and Choquet, 2020). The stabilization of actin filament by jasplakinolide reduces the mobility of the extrasynaptic AMPA receptor subunit GluA2, whereas destabilization of actin filament by latrunculin A results in increased movement of GluA2 subunits (Rust et al., 2010). This suggests that the effect of ADF/cofilin on AMPA receptors mobility and surface expression is mediated by actin-dependent mechanisms. These results are consistent with studies where direct manipulations of the actin cytoskeleton affects LTP and AMPA receptor trafficking (Allison et al., 1998; Kim and Lisman, 1999; Zhou et al., 2001; Cingolani and Goda, 2008; Yang et al., 2008; Hanley, 2014; Basu and Lamprecht, 2018). ADF/cofilin have also been shown to play a role in AMPA receptor internalization during LTD (Zhou et al., 2011). The induction of metabotropic glutamate receptor-dependent LTD (mGluR-LTD) induces ADF/cofilin dephosphorylation, spine shrinkage and a decrease in synaptic AMPA receptors. Actin-depolymerization factor/cofilin-dependent regulation of AMPA receptor trafficking is also seen following learning. Extinction of conditioned taste aversion leads to temporally enhanced ADF/cofilin activity in the infralimbic cortex of the rats and manipulations of ADF/cofilin activity accelerates or inhibits memory extinction by regulating the recruitment of AMPA receptors at the synaptic surface (Wang et al., 2013). These studies support that ADF/cofilin regulates synaptic transmission through AMPA receptor trafficking in addition to spine morphological changes.

### Mechanisms and Signaling Pathways Regulating ADF/Cofilin Activity During Synaptic Plasticity

**FIGURE 2:**
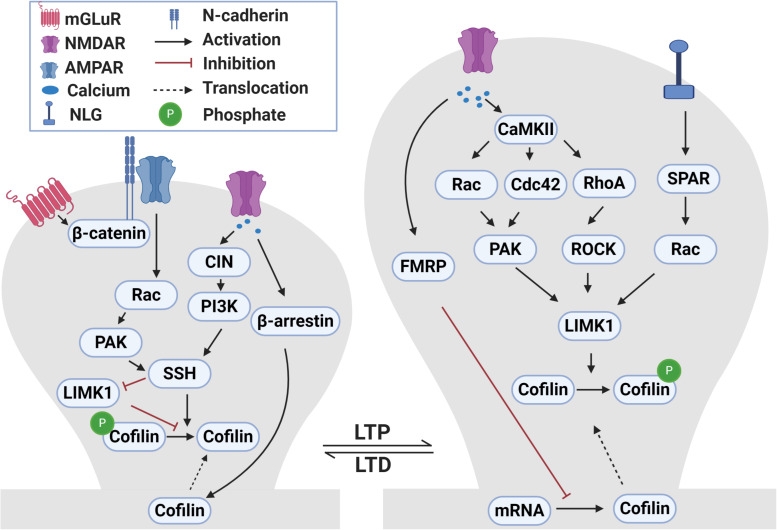
Signaling pathways that regulate ADF/cofilin phosphorylation and dephosphorylation during LTP and LTD. During LTP, activation of NMDA receptors causes calcium influx into dendritic spines. The increased intracellular calcium activates CaMKII which in turn activates small Rho GTPases, including Rac, Cdc42 and RhoA. These small GTPases bind to and activate PAKs and ROCKs that can directly phosphorylate and activate LIMK1. LIMK1 can also be activated following the activation of neuroligin 1 receptors during LTP through SPAR-Rac signaling pathway. Activated LIMK1 phosphorylates and inactivates cofilin resulting in the enlargement of dendritic spines. In addition, the LTP-induced calcium influx diminishes local translation of cofilin mRNA in dendrites through a FRMP 1-dependent manner. Translocation of cofilin into spines during LTP occurs through yet to be discovered mechanisms. During LTD, the activation of NMDA receptors and influx of calcium activates CIN which activates SSH through the PI3K-dependent pathway. Activated SSH dephosphorylates and activates cofilin which results in dendritic spine shrinkage. In addition, LTD-induced calcium influx mediates translocation of cofilin into spines in a β-arrestin 2-dependent manner. During mGLuR-LTD, GluA2 interaction with cadherin/β-catenin activates Rac-PAK which then activate SSH. Additionally, SSH can also dephosphorylate and inactivate LIMK1.

At many central synapses, the induction of LTP and LTD requires Ca^2+^-dependent signaling pathways, including protein kinases [e.g., activation of Ca^2+^/calmodulin-dependent protein kinase II (CaMKII) during LTP] and phosphatases (e.g., calcineurin) during LTD (Malinow and Malenka, 2002; Collingridge et al., 2004; Malenka and Bear, 2004; Derkach et al., 2007; Citri and Malenka, 2008; Lüscher and Malenka, 2012; Bliss and Collingridge, 2013; Huganir and Nicoll, 2013; Henley and Wilkinson, 2016; Sanderson et al., 2016; Diering and Huganir, 2018). Accumulating evidence indicates that multiple mechanisms exist at the synapse to link these Ca^2+^-dependent pathways to regulate ADF/cofilin (Meng et al., 2004; Jia et al., 2009; Rex et al., 2009; Martinez and Tejada-Simon, 2011; Yasuda, 2017; Nakahata and Yasuda, 2018) and these are summarized in Figure 2.

Many studies have shown that ADF/cofilin is a downstream effector of Rho GTPases and their effector protein kinases such as PAKs, ROCKs and LIMKs during LTP and spine enlargement (Borovac et al., 2018; Nakahata and Yasuda, 2018; Kovaleva et al., 2019). Rho proteins, including RhoA, Rac and Cdc42, are activated during LTP (Govek et al., 2005; Rex et al., 2009; Martinez and Tejada-Simon, 2011). For example, stimulation of NMDA receptors leads to activation of Rac1 and rapid enlargement of dendritic spines (Xie et al., 2007). Overexpression of either Rac1 or Rac3 causes an increase in spine density (Wiens et al., 2005; Pennucci et al., 2019). Double knockouts of Rac1 and Rac3 inhibit the formation of dendritic spines and induce an increase in filopodia-like spines (Pennucci et al., 2019). PAK1 and PAK3 double KO mice show decreased actin filaments and phosphorylated ADF/cofilin which are associated with immature spines and LTP impairments (Huang et al., 2011). Similarly, ROCK2 KO mice are altered in spine morphology accompanied by reduced phosphorylated ADF/cofilin (Zhou et al., 2009). LIMK1 KO mice exhibit significant abnormalities in the actin cytoskeleton, reduced phosphorylated ADF/cofilin and impaired late phase LTP (Meng et al., 2004; Todorovski et al., 2015). These genetic studies are consistent with results from manipulations of the Rho GTPases and their effectors in cultured neurons and slices (Luo et al., 1996; Nakayama et al., 2000; Tashiro et al., 2000; Rex et al., 2009; Shi et al., 2009). Therefore, ADF/cofilin phosphorylation mediated by the activation of the Rho GTPase-PAK/ROCK-LIMK pathway is a key mechanism that is responsible for ADF/cofilin inactivation, actin assembly and spine enlargement during LTP. Actin-depolymerization factor/cofilin dephosphorylation through activation of chronophin might also be important spine enlargement during LTP as chronophin KO mice are impaired in late-phase LTP (Kim et al., 2016). The effect of chronophin could be mediated through regulating the coupling of GluN2A subunits with postsynaptic proteins (Kim et al., 2016).

In addition to ADF/cofilin phosphorylation, reduced protein translation of ADF/cofilin has also been reported to be associated with chemical LTP and this translational regulation is mediated by fragile X mental retardation protein 1 (FMRP1; Feuge et al., 2019). In cultured hippocampal neurons, glycine induced LTP is accompanied by reduced ADF/cofilin mRNA availability and translation, and these changes are impaired in FMRP1 KO mice. How FMRP1-mediated suppression of ADF/cofilin translation is achieved remains unknown, but it is known that this mRNA-binding protein is a potent regulator of activity-dependent local protein synthesis involving the mTOR and ERK1/2 pathways (Brown et al., 2001), and therefore it is possible these pathways are also important for downregulating ADF/cofilin protein level during this form of LTP. Interestingly, the FMR1 KO mice also show elevated activation of the Rac-PAK-LIMK pathway, resulting in increased ADF/cofilin phosphorylation, under basal conditions, and overexpression of active ADF/cofilin rescues some of the behavior defects in FMR1 KO mice (Pyronneau et al., 2017). These results indicate that in addition to translational regulation of ADF/cofilin, FMRP1 also acts as a negative regulator of ADF/cofilin phosphorylation through the Rac-PAK/LIMK signaling process.

During LTD and spine shrinkage, Ca^2+^-dependent phosphatases are important for ADF/cofilin dephosphorylation and activation. Inhibition of calcineurin in CA1 pyramidal neurons blocks cofilin-dependent spine reduction during LTD induced by low frequency stimulation (Zhou et al., 2004). Chemical LTD induced by application of NMDA is associated with dendritic spine shrinkage and loss of synaptic proteins, and these changes require ADF/cofilin dephosphorylation and spine accumulation (Pontrello et al., 2012). Although calcineurin dependent activation of phosphatidylinositol 3-kinase (PI3K) is important for ADF/cofilin dephosphorylation, the intermediates between PI3K and ADF/cofilin are not yet identified. In non-neuronal cells, PI3K regulates cofilin dephosphorylation through activation of SSH (Nishita et al., 2004). Furthermore, calcineurin has been shown to mediate ADF/cofilin dephosphorylation by SSH in response to calcium influx (Wang Y. et al., 2005). In neuronal cells, ephrin-induced dendritic spine retraction and ADF/cofilin dephosphorylation requires activation of calcineurin and subsequent activation of SSH (Zhou et al., 2012). These studies suggest that the calcineurin-P13K-SSH pathway may mediate ADF/cofilin dephosphorylation and activation during LTD. The spine accumulation of ADF/cofilin during chemical LTD requires β-arrestin 2 as NMDA-induced spine remodeling and cofilin translocation are impaired in β-arrestin 2 KO neurons (Pontrello et al., 2012). The NMDA-induced cofilin dephosphorylation appears to be independent of spine accumulation as blocking the PI3K pathway does not prevent cofilin translocation to the spine. These results suggest that there are two distinct pathways respectively regulating cofilin dephosphorylation and spine accumulation that are activated during NMDA-induced spine shrinkage and LTD.

During mGluR-LTD, the mechanisms governing ADF/cofilin regulation are also distinct from those involved in NMDA receptor dependent LTD (Zhou et al., 2011). mGluR-LTD induces ADF/cofilin dephosphorylation and spine accumulation and these changes are required for both mGluR-dependent spine shrinkage and synaptic depression. Interestingly, ADF/cofilin dephosphorylation is dependent on the AMPA receptor subunit GluA2 and its interaction with the cell adhesion molecule N-cadherin/β-catenin and subsequent activation of Rac1. How the activation of Rac1 leads to dephosphorylation and activation of ADF/cofilin is not known, but it could involve inactivation of the PAK-LIMK pathway or activation of the SSH pathway.

In addition to glutamate receptors, other neuronal surface proteins and receptors have also been shown to regulate ADF/cofilin activity through similar mechanisms discussed above but may involve additional processes. For example, the c-terminal domain of the cell adhesion molecule neuroligin 1 induces spine enlargement and cofilin phosphorylation that are mediated by neuroligin 1’s interaction with spine-associated Rap GTPase-activating protein (SPAR) and subsequent activation of the Rac1-LIMK pathway (Liu et al., 2016). Neurotrophic factors and their receptors regulate spine growth and LTP which are dependent on changes in ADF/cofilin phosphorylation and dephosphorylation mediated by Rac1 and RhoA (Gehler et al., 2004; Soulé et al., 2006; Wong et al., 2019). Glucocorticoid hormone promotes learning-induced spine formation mediated by activation of LIMK1 and ADF/cofilin phosphorylation (Liston and Gan, 2011; Liston et al., 2013). These studies together indicate the complexity of the regulatory mechanisms governing ADF/cofilin activity at the synapse.

### Regulation of Presynaptic Function

Actin is also abundantly expressed in presynaptic terminals (Matus et al., 1982; Gotow et al., 1991). Pharmacological studies have identified a role for actin in regulating synaptic vesicle mobilization and exocytosis (Morales et al., 2000; Gorovoy et al., 2005). Like actin, ADF/cofilin are also expressed in presynaptic terminals (Rust, 2015), suggesting a presynaptic function. This is initially supported by alterations in presynaptic properties in LIMK1 KO mice where the frequency of neurotransmitter release and synaptic depression in response to sustained neuronal activity are both increased and (Meng et al., 2002). However, neurotransmitter release and presynaptic short-term plasticity are not affected in cofilin-1 KO mice (Rust et al., 2010). In addition, the recruitment and exocytosis of synaptic vesicles are unchanged in ADF KO mice (Görlich et al., 2011). The lack of presynaptic defects in ADF KO mice may be explained by the elevated cofilin-1 levels observed in these mice (Görlich et al., 2011). Indeed, ADF and cofilin-1 double KO mice have more severely impaired actin dynamics as well as altered distribution and exocytosis of synaptic vesicles (Wolf et al., 2015; Zimmermann et al., 2015). Electron microscopy and biochemical data from these double KO mice show a shift in the distribution from the active zone to the reserve pool as well increased docking of synaptic vesicles at CA1 synapse (Wolf et al., 2015). In addition, electron microscopy data from the double KO mice show an increase in the presynaptic bouton area and an increased number of docked vesicles at the active zone of striatal synapses, resulting in increased overall glutamate release at the striatal synapses (Zimmermann et al., 2015). Interestingly, a decrease in glutamate release is detected within the hippocampus and this decrease could be caused by defective vesicle recruitment as shown by reduced glutamate release during sustained synaptic stimulation (Wolf et al., 2015). Therefore, although cofilin-1 is a limiting factor in postsynaptic plasticity and cannot be substituted by ADF, the presence of either ADF or cofilin-1 appears to be sufficient to regulate actin remodeling during presynaptic vesicle release, suggesting an overlapping functions presynaptically (Rust et al., 2010; Wolf et al., 2015; Zimmermann et al., 2015). In line with the role of ADF/cofilin in presynaptic function, the disruption of upstream regulators, including RhoA, ROCK2, PAK1/3, LIMK1, and SSH, all impair some aspects of vesicle exocytosis and neurotransmitter release (Meng et al., 2002; Wang H. G. et al., 2005; Asrar et al., 2009; Yuen et al., 2010; Huang et al., 2011).

### Regulation of Learning and Memory

long-term potentiation and depression are regarded as key mechanisms for learning and memory (Bliss and Collingridge, 1993; Citri and Malenka, 2008; Neves et al., 2008; Kandel et al., 2014). The demonstrated role of ADF/cofilin in these forms of synaptic plasticity, as discussed earlier, suggests that they are important in memory formation and this is supported by several studies. For example, the conditional deletion of cofilin-1 in postnatal principal neurons results in severe impairments in associative learning, but not exploratory or latent learning (Rust et al., 2010). Activation and inhibition of ADF/cofilin activities using peptides facilitated or impeded contextual fear memory extinction in rats, respectively (Wang et al., 2013). Increased phosphorylated, inactive, ADF/cofilin is observed in the hippocampal CA1 region of rats after learning in an enriched environment (Fedulov et al., 2007). Neonatal social isolation inactivates ADF/cofilin and leads to an increase in stable actin fractions at the dendritic spines in the juvenile medial prefrontal cortex (Tada et al., 2016) and barrel cortex of rats (Tada et al., 2017). Other evidence supporting the importance of ADF/cofilin in memory comes from memory abnormalities observed in the absence of ADF/cofilin upstream regulators. Impaired learning has been documented for mice lacking LIMK1, PAK1/3 and Rho GTPases (Meng et al., 2002, 2005; van Galen and Ramakers, 2005; Huang et al., 2011; Todorovski et al., 2015). For example, LIMK1 KO mice are drastically impaired in long-term but not short-term memory during fear conditioning and the Morris water maze (Meng et al., 2002; Todorovski et al., 2015). Expression of dominant-negative PAK3 alters cofilin phosphorylation and impairs social recognition memory (Leung et al., 2018). Actin-depolymerization factor/cofilin also play a role in other behaviors including reward learning (Toda et al., 2006; Rothenfluh and Cowan, 2013) and anxiety (Goodson et al., 2012). Conditional cofilin-1 KO mice show impaired novel object recognition, but normal social behavior including social recognition (Sungur et al., 2018). Moreover, ADF/cofilin conditional double KO mice also demonstrate abnormal nesting behavior, increased activity and impulsive behavior, as well as reduced non-associative learning and working memory (Zimmermann et al., 2015).

## Role of ADF/Cofilin in Neuronal Apoptosis and Neuroinflammation

Translocation of ADF/cofilin to the mitochondria is important for induction of apoptosis in multiple cell types, including neurons, neutrophils, lymphoma, neuroblastomas, and prostate cancer (Chua et al., 2003; Zhu et al., 2006; Klamt et al., 2009). Actin-depolymerization factor/cofilin undergo oxidation during inflammatory stress (Klamt et al., 2009; Bernstein and Bamburg, 2010), and when oxidation is prevented, apoptosis is inhibited (Klamt et al., 2009). In mouse embryonic fibroblasts, oxidation of ADF/cofilin cause them to lose their affinity for actin and translocate to the mitochondria, where they induce swelling and cytochrome c release by mediating the opening of the permeability transition pore (Klamt et al., 2009). Knocking down endogenous ADF/cofilin using targeted small interfering (siRNA) inhibits apoptosis, which is restored by expression of wild type ADF/cofilin (Klamt et al., 2009). The apoptotic effect of ADF/cofilin is independent of ADF/cofilin’s role in actin cytoskeleton regulation (Bernstein and Bamburg, 2010).

Several studies have implicated ADF/cofilin in the regulation of neuronal apoptosis (Yang et al., 2004; Bernstein and Bamburg, 2010; Li et al., 2013). Knocking down ADF/cofilin from primary cortical neurons results in decreased excitotoxic neuronal death caused by excess glutamate (Posadas et al., 2012). During excitotoxic neuronal death, ADF/cofilin interacts with the proapoptotic protein Bax, carrying it to the mitochondria and contributing to the depolarization of the mitochondrial membrane, the release of apoptotic factors and neuronal death (Posadas et al., 2012). Actin-depolymerization factor/cofilin is also involved in ischemia-induced neuronal death (Madineni et al., 2016). The activation of ADF/cofilin occurs during ischemia in cortical neurons and knocking down ADFcofilin increases neuronal viability (Madineni et al., 2016). These results are consistent with work on LIMK1 and SSH (Yang et al., 2004; Posadas et al., 2012), showing that overexpression of LIMK1 and inhibition of SSH protects cells from apoptosis by inactivating ADF/cofilin.

In addition to direct involvement in neuronal apoptosis, recent studies suggest that ADF/cofilin contribute to neuronal apoptosis through other cell types like astrocytes (Alhadidi et al., 2016). Astrocytes express glutamate transporters which regulate the clearance of glutamate released from synapses (Anderson and Swanson, 2000). Dysfunction of astrocytic glutamate transporters trigger neuronal death by excessive glutamate and excitotoxicity (Rossi et al., 2000). In primary astrocyte cultures, the actin cytoskeleton has an important role in regulating the activity of glial glutamate transporters as inhibition of actin polymerization by cytochalasin-B reduces cell surface expression of these transporters (Adolph et al., 2007). Also, Rottlerin, a polyphenol natural product, decreases the activity of astrocyte glutamate transporters and disrupts actin filament dynamics (Sheean et al., 2013). Endocytosis of astrocyte glutamate transporters is also dependent on actin dynamics (Yan et al., 2014). These studies suggest that ADF/cofilin mediate actin changes and astrocytic glutamate transporters which in turn contribute to glutamate uptake and neuronal apoptosis.

ADF/cofilin have also been suggested to be involved in the regulation of neuroinflammation (Rasmussen et al., 2010; Gitik et al., 2014). Microglia and astrocytes are the first cells to be activated following brain injuries (Taylor and Sansing, 2013). In the Ra2 microglia cell line, ADF/cofilin knockdown inhibits nicotinamide adenine dinucleotide phosphate hydrogen (NADPH) oxidase activity and reactive oxygen species (ROS) formation which result in decline in cells activity (Rasmussen et al., 2010). Microglial cell phagocytic activity is also highly influenced by ADF/cofilin activation (Gitik et al., 2014). Similarly, ADF/cofilin activation is required for the restoration of the myelin sheath and involve in control of phagocytosis of degenerated myelin by microglia and macrophages (Hadas et al., 2012). Actin depolymerization by ADF/cofilin has also been shown to be important for exosome formation, which plays an essential role in facilitating neuroinflammation (Gupta and Pulliam, 2014).

## Role of ADF/Cofilin in Alzheimer’s Disease

Given the involvement of ADF/cofilin in the regulation of dendritic spines, synaptic plasticity and learning and memory, it is not surprising that deficits in ADF/cofilin are implicated in a wide range of brain disorders (Bamburg and Wiggan, 2002). These conditions include autism spectrum disorders (Duffney et al., 2015; Sungur et al., 2018), Williams syndrome (Hoogenraad et al., 2004), intellectual disability (Newey et al., 2005; van Galen and Ramakers, 2005; Zamboni et al., 2018), drug addiction (Rothenfluh and Cowan, 2013), sleep deprivation (Havekes et al., 2016) and neurodegenerative diseases such as AD (Liu et al., 2019), which will be discussed briefly below.

Alzheimer’s disease is a neurodegenerative condition characterized by memory loss and cognitive decline, resulting in the loss of independence and a shorter life span (Hsiao et al., 1996; Hsia et al., 1999; Li et al., 2014). Pathologically, AD is characterized by neurofibrillary tangles and senile plaques, consisting mainly of extracellular amyloid β (Aβ) peptides (Hsiao et al., 1996; Hsia et al., 1999; Hardy and Selkoe, 2002; Butterfield, 2014). In the brain, Aβ results from the proteolytic processing of the amyloid precursor protein (APP) and it has been proposed that the accumulation of toxic Aβ42 plays a major role in the development of dementia (Hardy and Selkoe, 2002; Palop and Mucke, 2010; Mucke and Selkoe, 2012). The effect of Aβ on the synapse and synaptic function, including LTP and LTD, is of great interest due to their direct relevance to learning and memory (Varadarajan et al., 2000; Selkoe, 2002; Lacor, 2007; Mucke and Selkoe, 2012; Sheng et al., 2012). Given the function of ADF/cofilin in synaptic plasticity, learning and memory, several studies have described the role of ADF/cofilin in the pathophysiology of AD. Actin-depolymerization factor/cofilin were discovered to accumulate in senile plaques in AD tissue and AD mouse models (Bamburg and Bernstein, 2016; Sun et al., 2019). Several studies show that brain tissue from AD patients and AD mouse models such as the APP/PS1 model exhibit elevated levels of inactive phosphorylated cofilin-1 (Barone et al., 2014; Gu et al., 2014; Han et al., 2017; Kang and Woo, 2019). On the other hand, multiple studies show that active dephosphorylated cofilin-1 forms aberrant cofilin-actin rods, which blocks axonal trafficking and may contribute to deficits in synaptic plasticity (Davis et al., 2011; Mendoza-Naranjo et al., 2012; Barone et al., 2014; Kang and Woo, 2019). Recently, it was shown that knocking down CAP2 in hippocampal neurons results in abnormal dendritic spines and impaired synaptic plasticity. This effect of CAP2 is relevant to ADF/cofilin because the CAP protein family is known to form a complex with ADF/cofilin and promote actin disassembly as discussed earlier. Moreover, chemical induction of LTP triggers CAP2 translocation to the spines and increases the formation of dimers, promoting the association of CAP2 with cofilin. In the hippocampal synapses of AD patients and mouse models, there is an increase in cofilin levels accompanied by a reduction in CAP2 synaptic availability, leading to a decrease in CAP2 dimer formation at the synapse (Pelucchi et al., 2020). Studies have also shown that the protein level of cofilin-2 is elevated in the brain and blood of AD patient brains and mouse models (Sun et al., 2015, 2019), but the significance of these changes will need further studies.

The disturbance in cofilin activity in AD may contribute to the loss of dendritic spines and synapses (Kang and Woo, 2019; Pelucchi et al., 2020). Decreased dendritic spine density and active synapses are seen in rat hippocampal pyramidal neurons from organotypic slices after exposure to Aβ oligomers (Shankar et al., 2007). Aβ-induced spine loss can be blocked by prevention of ADF/cofilin activation by expression of constitutively inactive cofilin (S3D) (Shankar et al., 2007). In the same line, Aβ42 oligomers promote ADF/cofilin dephosphorylation and activation in the hippocampus derived HT22 cell line and primary cortical neurons (Woo et al., 2015). Genetic reduction in ADF/cofilin activity activation rescues Aβ42-induced synaptic protein loss as well as deficits in LTP and contextual memory in APP/PS1 mice (Woo et al., 2015). These studies suggest the involvement of active cofilin in AD synaptic dysfunction. In addition, cofilin may also contribute to accumulation of Aβ aggregate and development of AD. For example, Aβ deposition in APP/PS1 mice is significantly decreased by genetically reducing cofilin using small interfering RNA (Liu et al., 2019). The effect of cofilin in Aβ accumulation may be through dual and opposing endocytic mechanisms promoting Aβ production in neurons and inhibiting Aβ clearance in microglia (Liu et al., 2019).

Despite the strong evidence for a role of ADF/cofilin dephosphorylation and activation in AD pathogenesis, several studies show that ADF/cofilin phosphorylation and inactivation may also play a role in AD pathogenesis (Kang and Woo, 2019). Acute exposure to Aβ oligomers increases the level of phosphorylated cofilin-1 at the postsynaptic compartment, leading to a subsequent stabilization of spine actin filaments, as well as impairment of chemically induced LTP (Rush et al., 2018). Also, Aβ oligomers increase cofilin phosphorylation and actin polymerization selectively in cholinergic basal forebrain neurons via increasing PAK1 phosphorylation and activity (Gu et al., 2014). There is also an increase in level of phosphorylated cofilin in synaptic fractions from APP/PS1 mice and AD patients’ brains (Rush et al., 2018). In APP/PS1 mouse brains, level of phosphorylated/inactive cofilin-1 is reduced at 4 months of age and increases at 10 months of age (Barone et al., 2014). In addition, different Aβ species and conformations seem to act differently on ADF/cofilin, depending on the locality, age and neuronal type (Kang and Woo, 2019). Despite this, the genetic reduction of ADF/cofilin rescues neurodegeneration (Woo et al., 2012), as well as LTP and contextual memory deficits in APP/PS1 mice (Woo et al., 2015). In summary, dysregulation of ADF/cofilin activity through either phosphorylation or dephosphorylation may contribute to the neurotoxic effects induced by Aβ in AD.

Several studies show that ADF/cofilin changes and synaptic dysfunction induced by Aβ are caused by both LIMK1 and SSH pathways (Kang and Woo, 2019). Treatment of hippocampal neurons with fibrillar amyloid beta increases the phosphorylation and activity of LIMK1 and these are accompanied by abnormalities in actin cytoskeleton, neuritic dystrophy and neuronal cell death (Heredia et al., 2006). Hippocampal neurons treated with Aβ42 oligomer induced LIMK1 activation which is regulated by an increase in the activity of Rac1 and Cdc42 Rho-GTPases and subsequent activation of PAK1 (Mendoza-Naranjo et al., 2012). Despite increased LIMK1 activation, Aβ42 treatments induce dephosphorylation of ADF/cofilin, suggesting the involvement of SSH. This is supported by work showing that overexpression of SSH prevents actin cytoskeleton abnormalities induced by Aβ42 treatments (Mendoza-Naranjo et al., 2012). In addition, Aβ42-induced ADF/cofilin dephosphorylation in the hippocampus-derived HT22 cell line is mediated by β1−integrin, a cell receptor important in the maintenance of synapses (Lilja and Ivaska, 2018), and the subsequent activation of SSH (Woo et al., 2015). These studies suggest that ADF/cofilin activity is regulated by bifurcating pathways that stimulate PAK1 and LIMK1 as well as SSH. In contrast, deficits in both PAK1 and PAK3 levels are detected in AD patients’ brains, which lead to activation of ADF/cofilin (Zhao et al., 2006). The application of Aβ42 can directly result in abnormally low levels of PAK in primary neurons (Zhao et al., 2006). Despite low levels of total PAK in AD brains, phosphorylated active PAK is increased around Aβ deposits (Zhao et al., 2006). Arsenault et al. (2013) confirm the loss of PAK in cortex of AD patients’ brains and cortex of AD mouse model (3xTg) and show that expression of a dominant-negative form of PAK results in deficits in social recognition.

In addition to the dysregulation of ADF/cofilin activity, formation of ADF/cofilin-actin rods may contribute to the pathology of AD (Bamburg and Bernstein, 2016; Kang and Woo, 2019). For example, an increase in ADF/cofilin-actin rods/aggregates have been reported in AD patients and AD mouse models including APP/PS1 and 3xTg (Rahman et al., 2014; Bamburg and Bernstein, 2016; Kang and Woo, 2019). Multiple studies have also shown that Aβ dimers/trimers promote the formation of ADF/cofilin-actin rods in neurons, associated with the dephosphorylation, activation of cofilin (Maloney et al., 2005; Davis et al., 2011; Mendoza-Naranjo et al., 2012; Barone et al., 2014). Bernstein et al. (2012) show that intermolecular disulfide bonds between cofilin subunits form *in vitro* by cofilin oxidation and is critical for cofilin-actin rod formation in stressed neurons. These less dynamic ADF/cofilin actin rods consist of ADF/cofilin and actin in 1:1 ratio and are shown to disrupt the integrity of dendritic microtubules, block intracellular transport of mitochondria and induce significant loss of dendritic spines (Bamburg and Bernstein, 2016; Walsh et al., 2014).

Targeting ADF/cofilin regulation may provide therapeutic targets to improve synaptic function and reduce memory impairment in AD patients (Shaw and Bamburg, 2017). Inhibition of cofilin-1 stabilizes the function and activity of dendritic spines in LTD mouse model (Zhou et al., 2004). Cofilin-1 inhibition is achieved using a phosphorylated peptide containing the first 16 amino acids of cofilin-1 (p-Cofilin peptide), which inhibits cofilin-1 activation through competitive binding to phosphatases. The use of this phosphorylated peptide in an AD mouse model (5 × FAD) rescues the deficits in surface expression and function of AMPA and NMDA receptors (Deng et al., 2016). Cofilin-1 inhibition by the peptide also partially improves working memory and novel object recognition in the model (Deng et al., 2016). In summary, ADF/cofilin contribute to AD pathology through multiple mechanisms including phosphorylation, dephosphorylation and formation of less dynamic ADF/cofilin-actin rods. Therefore, targeting ADF/cofilin holds promise to mitigate the physiological and behavioral abnormality in AD.

## Concluding Remarks

In summary, ADF/cofilin play multifaced roles in the regulation of synaptic structure and function in the brain. The temporal and spatial regulation of ADF/cofilin appears to be particularly important for the bi-directional effect on spine and synaptic plasticity. However, there are several key questions that need to be addressed further. First, although spine accumulation of ADF/cofilin is associated with both spine enlargement/LTP and LTD/spine shrinkage, how the increased ADF/cofilin in the spine leads to opposite changes in spine morphology remains unclear. Second, the relationship between spine accumulation and phosphorylation/dephosphorylation of ADF/cofilin needs further characterization. It remains unclear whether the translocation of the endogenous ADF/cofilin to the spine during LTP/spine enlargement or LTD/spine shrinkage requires ADF/cofilin dephosphorylation, although the exogenously expressed cofilin S3D appears unable to accumulate in the spine during LTP or LTD (Pontrello et al., 2012; Noguchi et al., 2016). Therefore, it is important to further elucidate how the translocation is regulated by protein phosphorylation and what protein kinases (e.g., LIMK1)/phosphatases (e.g., SSH) are involved. Third, the cooperation between ADF/cofilin and other actin-binding proteins (e.g., CAP2 and AIP) would provide another layer of regulation for ADF/cofilin activity at the synapse as some of these proteins also exhibit redistribution in the dendritic spine (Bosch et al., 2014; Pelucchi et al., 2020), but exactly when and how these interactions affect actin dynamics within the spine will require further studies. The use of photoactivatable ADF/cofilin (Noguchi et al., 2016; Senju et al., 2017; Borovac et al., 2018) or their upstream regulators (such as Rac1) (Wu et al., 2009; Fujii et al., 2013) in specific neuronal types and/or subcellular compartments within the spine should facilitate these investigations. Another emerging area is how ADF/cofilin-mediated actin dynamics are associated with and affect behavior in living animals, including different phases of learning and memory. As many brain disorders are associated with altered regulation of ADF/cofilin, a better understanding of this protein family could also aid in the understanding and treatment of these disorders.

## Author Contributions

All authors wrote and approved the manuscript.

## Conflict of Interest

The authors declare that the research was conducted in the absence of any commercial or financial relationships that could be construed as a potential conflict of interest.
